# Melissopalynological, physicochemical and antioxidant properties of honey from West Coast of Malaysia

**DOI:** 10.1007/s13197-019-03728-3

**Published:** 2019-04-03

**Authors:** Kirthiga Selvaraju, Paritala Vikram, Jan Mei Soon, Kumara Thevan Krishnan, Arifullah Mohammed

**Affiliations:** 1 Faculty of Agro Based Industry, Universiti Malaysia Kelantan, 17600 Jeli, Kelantan, Malaysia; 2 Faculty of Health and Wellbeing, University of Central Lancashire, Preston, UK

**Keywords:** Antioxidant, Malaysian honey, Phenolics, Stingless bees, Unifloral

## Abstract

Stingless bees are native to tropical region and produce honey which are high in moisture content. Compared to honey from honeybees, there are limited studies on honey derived from stingless bees. Hence, the aim of this study was to evaluate the chemical composition and antioxidant activities of stingless bee honey. Fifteen types of honey were collected from six states in West Coast of Malaysia and pollen analyses were carried out. Four types of unifloral honey samples produced by stingless bees were selected to determine their physicochemical and antioxidant activities including total phenolic, total flavonoid and ascorbic acid contents. Melissopalynological study of 15 honey samples collected from different states showed presence of both unifloral and multifloral origins. Honey samples collected from *Apis mellifera* (honeybee) combs had lower number of total pollen compared to samples collected from *Heterotrigona itama* and *Geniotrigona thoracica* (stingless bees). Jambul Merak honey contains the highest phenolic and flavonoid contents with greatest color intensity and has the highest antioxidant potential. This study highlights the chemical composition and biological activity of honey from stingless bees which may increase its commercial value or to be utilised as potential functional food ingredient.

## Introduction

Honey is a popular sugary product that is used as a natural sweetener in food and beverage products. Honey is essentially a supersaturated solution of monosaccharides in water. It also contains multiple components such as carbohydrates, amino acids, vitamins (Fechner et al. 2016), organic acids, esters, volatile components, hydroxymethylfurfural, enzymes and phytochemicals (Siddiqui et al. 2017). Honey from stingless bees contain higher amount of moisture and Marinus (2006) reported high water content of up to 42%. Stingless bees are native to Malaysia and are common to tropical and sub-tropical areas in South-east Asia region (Ramli et al. 2017). Honey composition can vary according to seasons, geographical area, entomological and floral sources (Singh and Bath 1997; Bath and Singh 1999), environmental, beekeeping and processing factors (Fechner et al. 2016; Vit et al. 2013; Hapburn and Radloff 2011).

Honey is widely consumed as a health product, but due to its high commercial value, it is often adulterated or labelled falsely for economic gains (Siddiqui et al. 2017). Although there exist methods to determine the botanical origin of honey using color, taste, flavour and content of physiologically active ingredients, Kaškonienė et al. (2010) argued that the information obtained by these tests are not always a reliable determination of botanical origin of honey. Other advanced analytical methods such as inductively coupled plasma mass spectrometry (ICP-MS) (Batista et al. 2012) and nuclear magnetic resonance spectroscopy (Consonni et al. 2013) had been utilised to determine the geographical origin of honeys.

Melissopalynological studies had been widely used to determine the geographical and floral origin of honey (Rodopoulou et al. 2018). Honey consists of pollen grains collected by honeybees (Shubharani et al. 2012) hence; pollen analysis of honey will assist in the identification of plant species origin. The identification of different plant species is vital as they contribute towards the composition of honey (Sodre et al. 2007) and helps to verify honey authenticity. Honey is classified into unifloral or multifloral honey. Unifloral honey consists of nectar collected from one type of floral source. Unifloral honey contains distinct flavour and color reflecting the type of flowers from which the nectar is collected (Agashe and Caulton 2009). Unifloral honeys like Manuka, Tualang, jujube and acacia are highly valued but easily adulterated due to their higher price (Sun et al. 2017). There are also more studies conducted on honey from honey bees compared to stingless bees. Thus, the current study aims to to evaluate the chemical composition and antioxidant activities of stingless bee honey.

## Materials and methods

### Honey samples

Fifteen honey samples (250 g each) were collected during dry season (March–April 2014) from apiaries in six different states of West Coast of Malaysia namely, Kedah (Kubang Pasu, and Padang Terap), Perak (Bukit Merah, and Kuala Kangsar), Melaka (Ayer Keroh, Hulu Melaka and Jasin), Negeri Sembilan (Tampin), Johor (Segamat), and Penang (Gertak Sanggul, Relau, Kaw District, Sungai Lembu 1, Sungai Lembu 2 and Sungai Lembu 3). All samples were subjected to melissopalynological analyses.

### Melissopalynological analyses

The honey samples were acetolysed to analyse the presence of different pollen. Acetolysis refers to the acidic hydrolysis/acetylation of pollen grains to remove the protoplasmic contents leaving only pollen with its exine layer for examination (Erdtman 1960). The acetolysed pollens were mounted into glycerin jelly (Agashe and Rangaswamy 1997) and placed onto microscopic slides for the examination.

### Pollen count

**Fig. 1:**
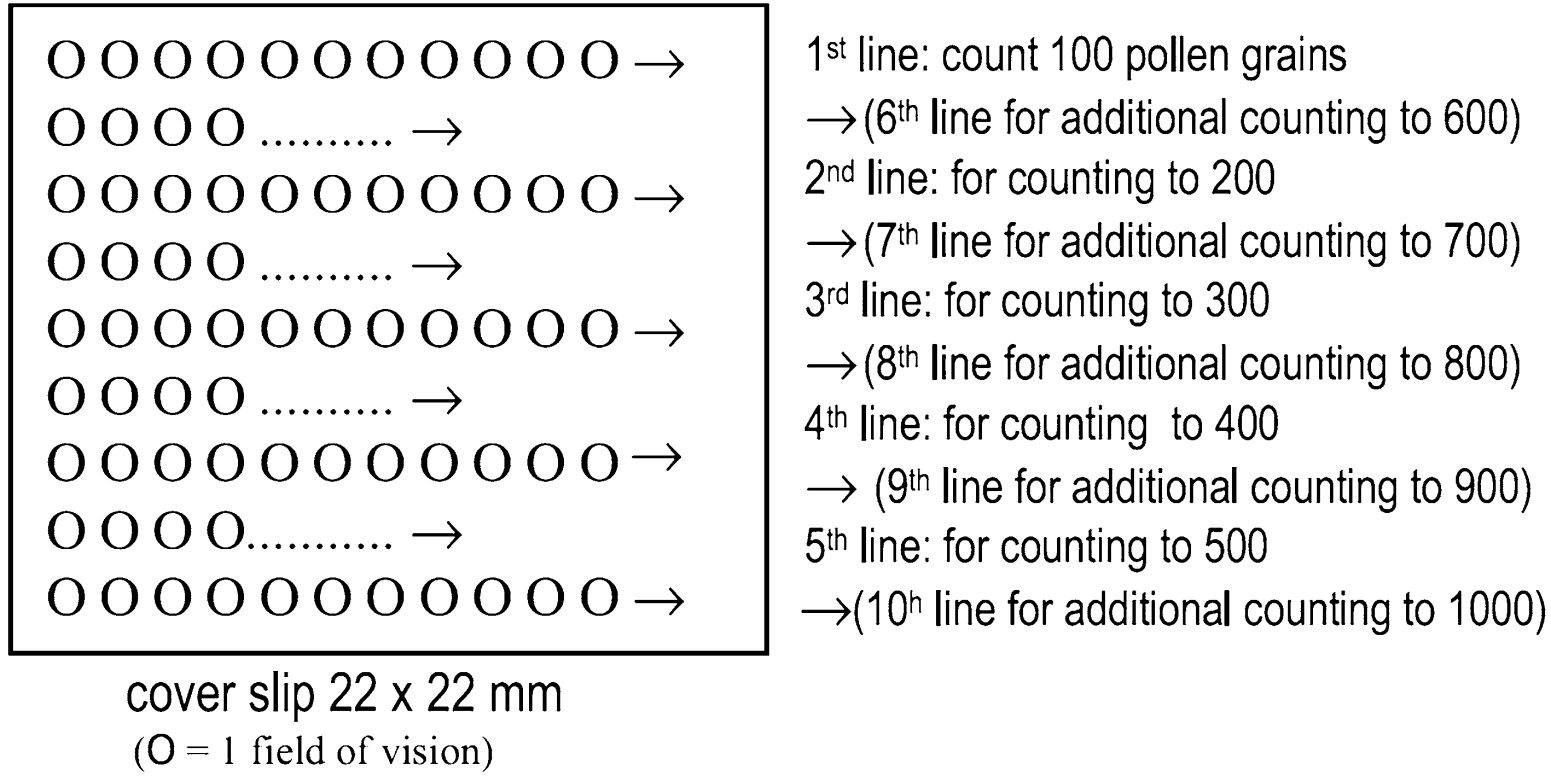
Matrix for counting pollen grains that guarantees a homogeneous examination of the slide (Ο = a whole microscopic field of view) (Ohe et al. 2004)

In order to determine pollen types and densities, pollen counts were performed according Ohe et al. (2004). This method identifies and counts pollens in groups of 100 according to the five parallel uniform equidistant lines from one edge of the cover slip until the other edge (Fig. 1).

The following equation (Eq. 1) was used to obtain the total pollen count per slide. Square coverslip = 50 views per side.

$$\begin{aligned} {\text{Area}}\;{\text{of}}\;{\text{coverslip}} & = L \times L \\ & = 50 \times 50 \\ & = 2500 \\ \end{aligned}$$

1 $${\text{Total}}\;{\text{pollen}}\;{\text{count}}\;{\text{per}}\;{\text{slide}} = N \times \frac{2500}{10}$$

where N represents the number of pollen counted in a slide.

2 $${\text{Percentage}}\;{\text{of}}\;{\text{abundance}},\;\% = \frac{{{\text{Total}}\;{\text{number}}\;{\text{of}}\;{\text{pollen}}\;{\text{of}}\;{\text{a}}\;{\text{particular}}\;{\text{species}}}}{{{\text{Total}}\;{\text{number}}\;{\text{of}}\;{\text{observed}}\;{\text{pollen}}}} \times 100.$$

### Physicochemical analyses

#### Chemicals and reagents

2,2-Diphenyl-1-picrylhydrazyl (DPPH), 2,6-dichlorophenolindophenol (DCPIP), butylated hydroxytoluene (BHT), Folin–Ciocalteu’s reagent, L-ascorbic acid, catechin, gallic acid and metaphosphoric acid were purchased from Sigma-Aldrich (St. Louis, MO, USA). Sodium carbonate (Na_2_CO_3_), aluminum chloride (AlCl_3_), sodium nitrite (NaNO_2_), glucose, glacial acetic acid (CH_3_COOH), potassium hydroxide (KOH), gelatin powder, glycerine, safranin powder, phenol crystals. Sodium hydroxide (NaOH) were purchased from Merck (Darmstadt, Germany). All chemicals used were of analytical grade.

#### pH and color

Ten percent (w/v) honey solutions were prepared using distilled water and pH was measured using a pH probe meter. The color was measured as described in Moniruzzaman et al. (2013a). Briefly, homogenised honey samples were transferred into a cuvette and colors were measured using color photometer. The color grades were expressed in millimeter (mm) Pfund grades against glycerol standard. Measurements were done using approved color standards (USDA 1985). The color intensity of honey samples was measured by first preparing 50% (w/v) of honey solution in warm distilled water (45–50 °C), filtered with 0.45 μm filters and color intensity was determined using spectrophotometer (Ferreira et al. 2009; Lacerda et al. 2010).

#### Sugar content

Twenty-five percent (w/v) honey solution was prepared and a visual handheld refractometer (ATAGO, N-1α, Tokyo, Japan) was used to determine the total sugar content of each honey sample. The percentage of sucrose content was measured in g/100 g of honey. The total reducing sugar content was measured using 3, 5-dinitrosalicylic acid (DNSA) according to Moniruzzaman et al. (2013a).

#### Ascorbic acid content

One hundred milligrams of honey was diluted with 10 ml of 1% metaphosphoric acid, filtered and mixed with 9 ml of 0.005% 2, 6-dichlorophenolindophenol and the absorbance of the mixture was measured at 515 nm within 30 min as described by Ferreira et al. (2009). The ascorbic acid content was calculated based on a calibration curve of pure L-ascorbic acid (50, 100, 200 and 400 μg/mL; Y = 3.2453X–0.0703; r^*2*^ = 0.9440).

### Analysis of antioxidant properties

The total phenolic content (TPC) of the honey sample was determined using spectrophotometric Folin–Ciocalteu method (Singleton et al. 1999). Ten mL honey solution was prepared by suspending 2 g of honey in distilled water and then 1 mL (0.2 g/mL) of honey solution was fortified with 1 mL of Folin and Ciocalteu’s phenol reagent. After incubating for 3 min, 1 mL of Na_2_CO_3_ solution (10%) was mixed and volume was made up to 10 mL with distilled water. The absorbance was recorded after reaction mixture was left in the dark for 90 min at 725 nm using a UV/VIS spectrophotometer. Standard curve was prepared using gallic acid.

The total flavonoid content (TFC) was determined using colorimetric assay as described in Zhishen et al. (1999). Firstly, 2 g of honey was suspended in distilled water and made up to 10 mL. 1 mL of suspension was taken and mixed with 4 mL of distilled water. After that 0.3 mL of NaNO_2_ (5% w/v) was gently added. After 5 min of incubation, 0.3 mL of 10% AlCl_3_ was mixed and incubated for 6 min. Later the mixture was added with 2 mL of 1 M NaOH and the volume was made up to 10 mL using distilled water. The reaction mixture was thoroughly shaken before reading the absorbance at 510 nm. A standard calibration curve was made using various dilutions of catechin.

The antioxidant potentials of the unifloral honey were investigated using the free radical-scavenging activity of the DPPH. 10 mL suspension was made by suspending 2 g of honey in distilled water. 0.5 mL of honey suspension was fortified with 2.7 mL of methanolic DPPH solution (0.024 mg/mL). The reaction mixture was thoroughly mixed, incubated for 15 min in the dark and the absorbance of the mixture was read at 517 nm. The reduction of the DPPH radical was calculated as described by Moniruzzaman et al. (2013a).

### Statistical analyses

Analysis of variance (ANOVA) was carried out using SPSS (version 22.0, SPSS Inc. IL). Determination of significant differences between honey samples were carried out using Tukey’s HSD post hoc tests (*p* ≤ 0.05).

## Results and discussion

### Pollen analysis

**Fig. 2:**
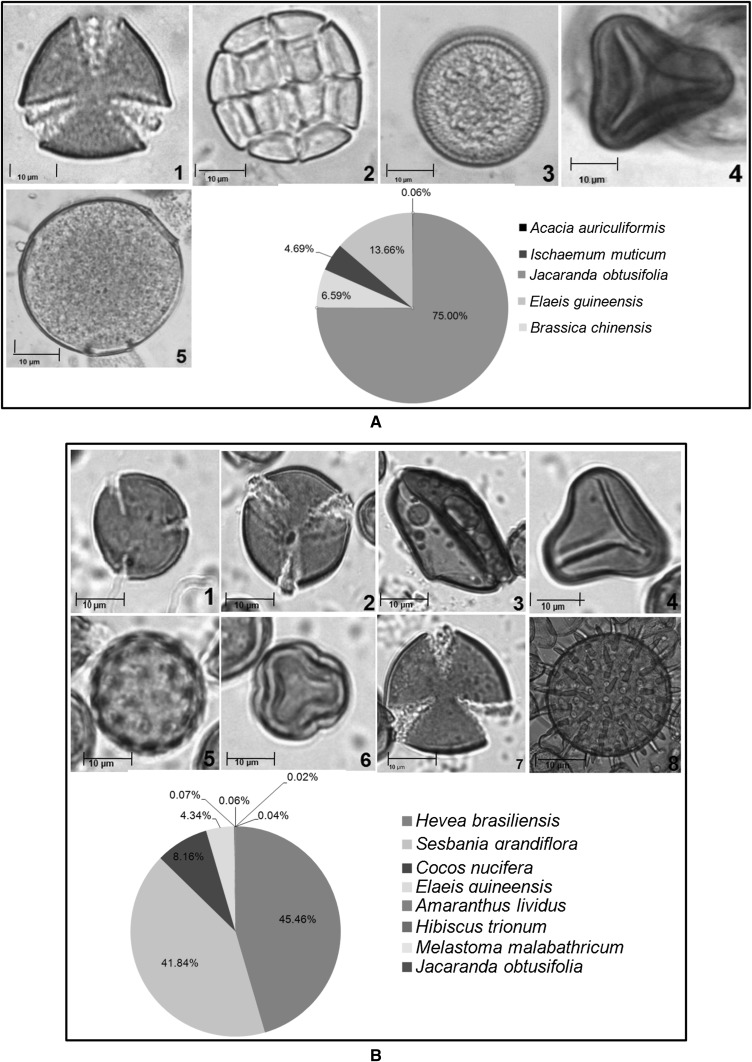

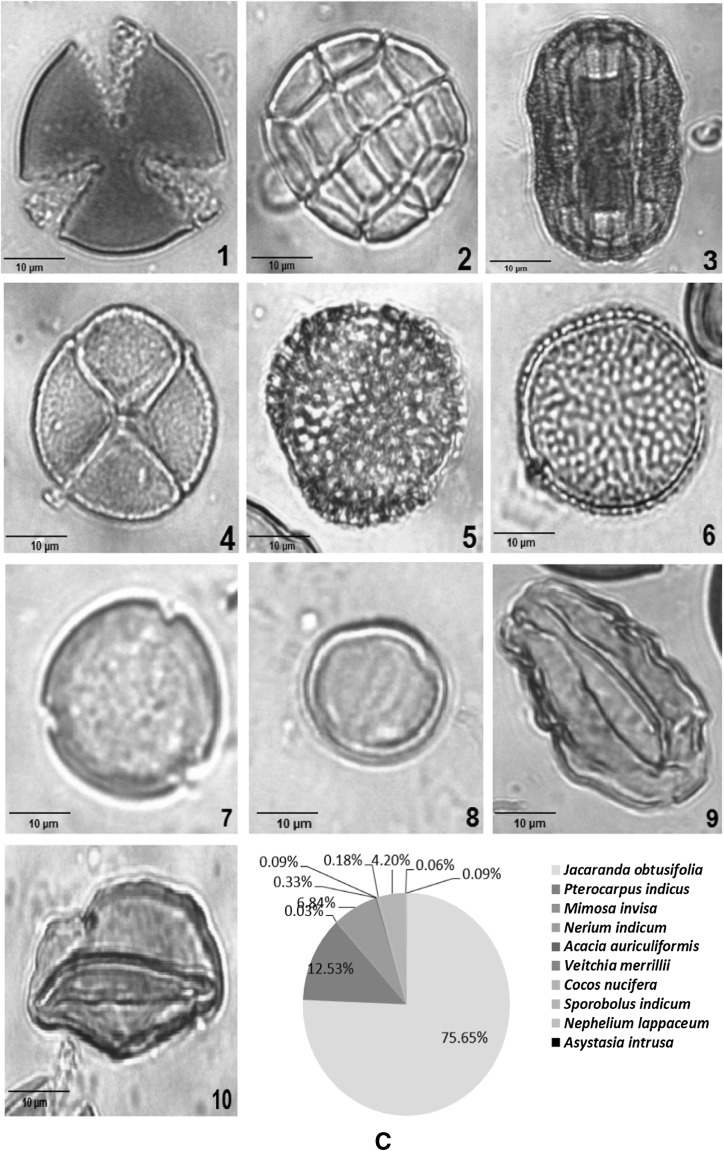

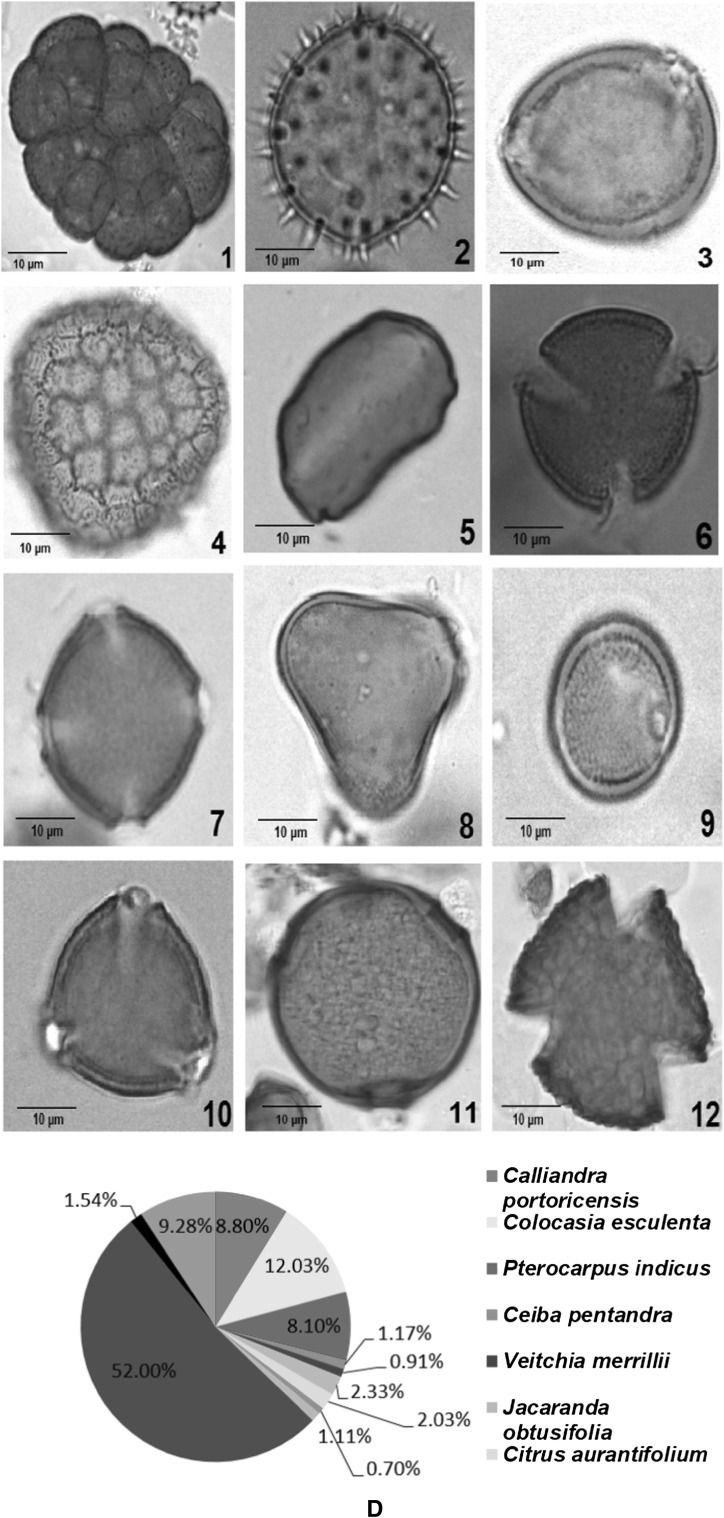
Melissopalynological analyses of unifloral honey of singles bees. Pollen types of **a** Kubang Pasu, Kedah (KPK), **b** Jasin, Melaka (JSM), **c** Padang Terap, Kedah (PTK), **d** Gertak Sanggul, Penang (GSP)

Melissopalynological study of 15 honey samples collected from different states of West Coast of Malaysa showed presence of both unifloral and multifloral origins (Table 1). Honey samples collected from *Apis mellifera* (honeybee) combs had lower number of total pollen compared to samples collected from *Heterotrigona itama* and *Geniotrigona thoracica* (stingless bees). This may be due to the size of *stingless* bees which are smaller compared to *Apis mellifera* bees which enables them to enter the deepest spaces of flowers (Kiew and Muid 1991). Furthermore, the flower morphology determines the accessibility to floral resources, as stingless bees and honey bees with different tongue length could affect their accessibility and feeding on nectar from different plant species (Hrncir and Silva 2013). In the present study, all the unifloral honeys—KPK, PTK, JSM and GSP were from *Heterotrigona itama* and *Geniotrigona thoracica* (Fig. 2). Sixty types of species from 34 different families were identified along with 4 unidentified samples (Table 2). The families *Leguminosae, Bignoniaceae, Cruciferae, Arecaceae* and *Gramineae* were represented in more than half of the samples. *Brassica bertonensis, Elaeis guineensis*, *Cocos nucifera* and *Jacaranda obtusifolia* were widely present in more than 50% of the samples (Table 2). The honey sample from JSM had the highest number of pollen (58,994) and sample from TPN had the least number of pollen compared to other honey samples collected. Although honey samples from TPN had 12 different species of pollen and SGJ had 11 species of pollen (Table 2), both honey samples had the lowest total number of pollen count which was 53 and 58 respectively compared with the other samples (Table 1).

**Table 1:** Total number of pollens according to their areas of origin and types of honey

Locality	State	Bee species	Total no. of pollen per slide	Classification of honey
Kubang Pasu (KPK)	Kedah	*Heterotrigona itama*	39,998	Unifloral
Padang Terap (PTK)	Kedah	*Heterotrigona itama*	3289	Unifloral
Bukit Merah (BMP)	Perak	*Heterotrigona itama*	52,686	Multifloral
Kuala Kangsar (KKP)	Perak	*Heterotrigona itama*	43,973	Multifloral
Ayer Keroh (AKM)	Melaka	*Geniotrigona thoracica*	27,650	Multifloral
Hulu Melaka (HMM)	Melaka	*Geniotrigona thoracica*	32,295	Multifloral
Jasin (JSM)	Melaka	*Geniotrigona thoracica*	58,994	Unifloral
Tampin (TPN)	Negeri Sembilan	*Apis mellifera*	53	Multifloral
Segamat (SGJ)	Johor	*Apis mellifera*	58	Multifloral
Gertak Sanggul (GSP)	Penang	*Heterotrigona itama*	30,621	Unifloral
Relau (RLP)	Penang	*Heterotrigona itama*	32,768	Multifloral
Kaw District, Bukit Mertajam (KBP)	Penang	*Apis mellifera*	71	Multifloral
Sungai Lembu 1 (LP1)	Penang	*Apis mellifera*	191	Multifloral
Sungai Lembu 2 (LP2)	Penang	*Apis mellifera*	485	Multifloral
Sungai Lembu 3 (LP3)	Penang	*Apis mellifera*	257	Multifloral

**Table 2:** Pollen profile of different types of honeys from different places of Malaysia (%)

	KPK	PTK	BMP	KKP	AKM	HMM	JSM	TPN	SGJ	GSP	RLP	KBP	LP1	LP2	LP3
Acanthaceae															
*Asystasia intrusa*		0.03													
*Pseuderanthemum variabile*											9.67				
Amaranthaceae															
*Achyranthes velutina*					8.72										
*Amaranthus lividus*			4.08	1.86			0.07					1.41			1.17
Anacardiaceae															
*Anacardium occidentale*											0.19				
Araceae															
*Alocasia brisbanensis*					10.24										
*Colocasia esculenta*			2.96		2.24					12.03					
Arecaceae															
*Areca catechu*											14.58				
*Cocos nucifera*		0.33	0.09	3.08	13.2	10.79	8.16	3.77			0.10	7.04	2.62		
*Elaeis guineensis*	13.66			2.61	12.75	3.35	4.34	3.77			0.03	2.82	2.62	0.21	1.17
*Veitchia merrillii*		0.09	0.11			14.93		3.77		0.91					
Apocynaceae															
*Nerium indicum*		6.84													
*Thevetia peruviana*												5.63			6.23
Asteraceae															
*Bidens pilosa*				5.78				9.43	3.45					0.62	10.89
*Cichorium pumilum*									1.72						0.39
*Pluchea indica*									1.72						
Bignoniaceae															
*Jacaranda obtusifolia*	75.00	75.65	7.85	20.81			0.04	28.30		2.33				1.65	20.23
*Tecomaria capensis*												14.08	13.61	37.94	
*Tecoma stans*								26.42							
Bombacaceae															
*Ceiba pentandra*			3.73							1.17					
*Durio zibethinus*										1.54	0.48				
Casuarinaceae															
*Casuarina equisetifolia*					5.15										
Cucurbitaceae															
*Cucumis melo*										52.00					
*Cucumis sativus*															3.11
Cruciferae															
*Brassica chinensis*	6.59		0.16	1.07	2.71			5.66				11.27		1.86	9.73
Cyperaceae															
*Cyperus brevifolius*													32.98		
Dilleniaceae															
*Dillenia suffruiticosa*			43.46	27.05											
Euphorbiaceae															
*Hevea brasiliensis*					4.58		50.46								
*Manihot esculenta*			6.09	1.65	0.45				13.79						
Gramineae															
*Ischaemum muticum*	4.69				21.58	19.86									
*Sporobolus indicum*		4.20	3.58	6.85						1.11					
*Zea mays*				2.93				11.32							
Labiatae															
*Ocimum basilicum*								1.89							
Leguminosae															
*Acacia auriculiformis*	0.06	0.18													
*Calliandra portoricensis*										8.80					
*Cassia biflora*									3.45						
*Leucaena leucocephala*									32.76			36.62		22.47	40.47
*Mimosa invisa*		0.09		0.08											
*Pterocarpus indicus*		12.53				25.99				8.10					
*Sesbania grandiflora*							36.84								
Malvaceae															
*Hibiscus trionum*							0.02	1.89	12.07						
Melastomataceae															
*Melastoma malabathricum*							0.06								
Moringaceae															
*Moringa pterygosperma*			3.18	1.33											
Musaceae															
*Musa*									15.52						
Myrtaceae															
*Baeckea crassifolia*				22.65	8.80	8.15				0.70					
*Psidium guajava*															1.17
Passifloraceae															
*Passiflora aurantia*								1.89			0.20				
*Passiflora edulis*					1.33										
Pedaliaceae															
*Sesamum orientale*											3.92		8.38		
Poaceae															
*Triticum monococcum*									5.17						
Polygonaceae															
*Antigonon leptopus*									8.62			15.49	13.61	33.61	
Rutaceae															
*Citrus aurantifolium*					3.96			1.89		2.03	14.83				
Sapindaceae															
*Nephelium lappaceum*		0.06			4.30	16.93									
Sapotaceae															
*Mimusops elengi*				2.24											
Solanaceae															
*Solanum torvum*									1.72						
Tiliaceae															
*Muntingia calabura*														0.62	
Urticaceae															
*Urtica dioica*			24.70												
Verbanaceae															
*Clerodendrum inerme*											29.06				
*Vitex lucens*										9.28	26.97		16.75		
Unknown 1												5.63			
Unknown 2													9.42		
Unknown 3														1.03	
Unknown 4															5.84

Pollen of a particular plant species is said to be predominant if its occurrence in the honey sample is more than 45% of total pollen count (Agashe and Caulton 2009). The predominant pollen in the honey samples (KPK) and Padang Terap (PTK) was *Jacaranda obtusifolia* measuring 75% (Table 2; Fig. 2). Therefore, this honey sample is classified as unifloral since the predominant taxon exceeds 45%. Stingless bees preferred to forage the Jacaranda plant which flowers intermittently throughout the year in Kubang Pasu and Padang Terap districts (Table 1).

The predominant pollen found in honey sample from Jasin, Melaka was *Hevea brasiliensis,* measuring 50.46% of the overall pollen count (Table 2; Fig. 2). *Hevea brasiliensis* (rubber tree) is locally known as ‘Pokok Getah’ in Malay. Malaysia is one of the top rubber producer in the world and the large cultivated area provide stingless bees with multiple nectar source. Rubber tree honey is also produced by *Apis mellifera* bees (Moniruzzaman et al. 2017). The predominant pollen in honey collected from Gertak Sanggul was *Cucumis melo* (honey dew melon or musk melon) which accounted for 52% of the total pollen count (Table 2; Fig. 2). *Cucumis melo* is an annual, herbaceous creeping plant most widely cultivated for its fruits. Bee species like *Apis mellifera, Hypotrigona para* and *Tetragonula carbonaria* are considered potential pollinators and the stingless bees collect both pollen and nectar (Kouonon et al. 2009).

Bees collect pollen and nectar according to the availability of botanical resources within their foraging ranges which are affected by the environmental and seasonal factors (Fechner et al. 2016). In the current study, almost all honey samples contained pollen from *Cocos nucifera* and *Elaeis guineensis*. The local honeybees and stingless bees have preference to forage these plants as they flower throughout the year and moreover, *Cocos nectar* have higher sugar concentrations. All honeys have their own markers and the pollens of *Cocos nucifera* and *Elaeis guineensis* are one of the most important markers to distinguish Malaysian honey from imported honeys of Australia or even China (Kiew and Muid 1991). This reiterates the importance of pollen analysis in determining the botanical and geographical origin of honeys. The higher pollen count found in honey of stingless bees could also be due to the higher presence of stingless bees which also predate the stinging honey bee (Ramli et al. 2017).

### Physicochemical analyses of unifloral honeys

#### pH

The pH influences honey texture, stability and shelf life and a pH value between 3.4 and 6.1 indicates the freshness of honey samples (Khalil et al. 2012). The pH values of all honey samples in this study range from 3.22 to 4.03 (Table 3). The pH values of unifloral Malaysian honey samples were similar to those previously reported in Malaysian honeys (between pH 3.49 and 4.70) (Moniruzzaman et al. 2013a, b). Jambul Merak honey from Padang Terap was the most acidic (3.22 ± 0.01). The fermentation of sugar into organic acids by yeasts contributes to the high acidity of honey (Ajlouni and Sujirapinyokul 2010). The variations in pH values of Jambul Merak honey from different locations are due to the different geographic origins as the nectar’s pH and soil conditions may influence honey physicochemical properties (Khiati 2014).

**Table 3:** Physical and chemical parameters of unifloral honey samples

Honey sample (location)	pH	Color intensity ABS_450_ (mAU; 50 w/v)	Total sugar content (%) (g/100 g of honey)	Reducing sugar (%) (g/100 g of honey)	Sucrose (%) (g/100 g of honey)	Ascorbic acid content (mg of ascorbic acid/kg of honey)
Honeydew (Gertak Sanggul)	4.03 ± 0.01^a^	512.00 ± 1.00^d^	70 ± 0.00^a^	92 ± 0.00^a^	16 ± 0.00^a^	6.69 ± 0.19^a^
Rubber Tree (Jasin)	3.35 ± 0.00^c^	778.33 ± 0.58^c^	67 ± 0.00^b^	90 ± 0.01^b^	15 ± 0.00^b^	3.60 ± 0.38^b^
Jambul Merak (Kubang Pasu)	3.42 ± 0.01^b^	1141.33 ± 1.15^a^	62 ± 0.00^c^	47 ± 0.00^c^	15 ± 0.00^c^	2.02 ± 0.19^c^
Jambul Merak (Padang Terap)	3.22 ± 0.01^d^	950.00 ± 2.00^b^	54 ± 0.00^d^	40 ± 0.00^d^	14 ± 0.00^d^	1.13 ± 0.37^d^

In each column, values with different letters (superscripts) indicate significant differences (*p* < 0.05) (mean ± SD)

#### Color characteristics

Color is the primary characteristic of honey and can be used in the identification of its floral origin (Bertoncelj et al. 2007) and is classified according to USDA-approved color standards (USDA 1985). The color of honey ranges from light yellow to dark amber with the occasional green or red hues. There are also black colored honey in extreme cases (Diez et al. 2004). The color of honey depends on the contents of water, saccharides, minerals, pollens, and polyphenolic compounds (Lachman et al. 2010). Jambul Merak honey from Kubang Pasu and Padang Terap and Rubber Tree honey from Jasin were classified as amber according to the USDA-approved color standards. They also exhibited the highest Pfund value (89, 86 and 88 respectively). Honey Dew honey from Gertak Sanggul is light amber in color and had lower Pfund value (75). The higher Pfund values may indicate higher antioxidant potential and the presence of different pigment compounds, such as phenolic compounds, flavonoids and carotenoids. Higher antioxidant activity had been reported in dark colored honeys (Aljadi and Kamaruddin 2004). Honey also darkens more rapidly when stored at high temperature and the length of storage time affects the honey’s color (Belay et al. 2015).

#### Color intensity ABS_450_

Color intensity of honey has a strong positive correlation with their antioxidant activities (Saxena et al. 2010). The color intensities of the investigated honey samples ranged between 512 and 1141.33 mAU (Table 3). Jambul Merak honey from Kubang Pasu had the highest color intensity (1141.33 ± 1.15 mAU). The color intensity of other honey samples were much lower (512–950 mAU), indicating lower potential antioxidant properties. The color intensity of the honey sample is represented by the ABS_450_ which is also commonly correlated with the phenolic and flavonoid content of the honey (Moniruzzaman et al. 2013c; Bertoncelj et al. 2007). The strong and positive correlation observed between total phenolic content and color intensity of honey samples suggests that honey with darker color contains higher total phenolic content (Kek et al. 2014). This is also reflected in this study where honey samples with higher phenolic and flavonoid contents tend to have significantly higher color intensities, as observed with Jambul Merak honey from Kubang Pasu.

#### Total sugar, reducing sugar and sucrose content

The major sugars present in honey are fructose and glucose along with sucrose in minor quantities (Ajlouni and Sujirapinyokul 2010). The mean total sugar content of honey samples ranged from 54 to 70% (Table 3). The total sugar content of Honey Dew honey, Rubber Tree honey and Jambul Merak honey from Kubang Pasu were between 62.00 and 70.00 g/100 g. These were above the limit for total sugar content (≥ 60%) as determined by the European Community directive (Council Directive 2001/110/EC 2002). The differences in total sugar content of Jambul Merak honey from Kubang Pasu and Padang Terap (*p* < 0.05) indicated that geographical origin also influence the total sugar content of honey (Moniruzzaman et al. 2013a). The reducing sugar content of both Jambul Merak honeys were between 40 and 47%. This might be due to the conversion of sugars into organic acids (Cavia et al. 2007). The sucrose contents of the tested unifloral honey samples ranged from 14 to 16%. According to Moniruzzaman et al. (2013b), the variations in the sucrose levels may be indicative of the effect that different geographical origin have on the compositional differences of honey.

#### Ascorbic acid content

Honey consists of mixtures of antioxidant compounds such as ascorbic acid and enzymes for instance glucose oxidase and catalase. The honey samples have ascorbic acid contents ranging from 1.13 to 6.69 mg/kg (Table 3). The variations in ascorbic acid content among the honey samples might be due to different geographical origins and nectar sources (Moniruzzaman et al. 2013a). Honey contains ascorbic acid because most flowers on which the bees forage contain this vitamin. It has been shown that antioxidant activity of honey, which depends on its nectar sources, is related to its vitamin C contents which has a significant impact on total antioxidant activity of honey (Buba et al. 2012).

### Antioxidant studies

The total phenol content of the honey samples ranged from 65.67 to 114.38 mg gallic acid/kg of honey (Table 4). The variations in phenolic content among the honey samples may be due to the different geographical and nectar sources of the honey. Other factors include collection season, storage and processing conditions (Kek et al. 2014). Phenolic compounds such as gallic, coniferic, benzoic and trans-cinnamic acids were detected in Malaysian honeys by Moniruzzaman et al. (2017). Jambul Merak honey (KPK) showed the highest amount of TPC (114.38 mg gallic acid/kg) indicating its high antioxidant potential.

**Table 4:** Antioxidant parameters of unifloral honey samples

Honey Sample (Location)	Total phenolics (mg gallic acid/kg)	Total flavonoids (mg catechin/kg)	Percentage of radical scavenging activity (%)
Honeydew (Gertak Sanggul)	65.67 ± 0.38^d^	10.18 ± 1.04^b^	27.12 ± 1.20^d^
Rubber Tree (Jasin)	74.11 ± 0.31^c^	11.77 ± 0.80^ab^	50.37 ± 0.98^c^
Jambul Merak (Kubang Pasu)	114.38 ± 1.05^a^	12.68 ± 0.14^a^	67.66 ± 0.87^a^
Jambul Merak (Padang Terap)	91.32 ± 0.36^b^	10.27 ± 0.29^b^	58.71 ± 0.78^b^

In each column, values with different letters (superscripts) indicate significant differences (*p* < 0.05) (mean ± SD)

The total flavonoid content of the honey samples were obtained using colorimetric assay. Similar to the polyphenol content, Jambul Merak (KPK) honey contained the highest amount (12.68 mg catechin/kg of honey) of flavonoids followed by Rubber Tree honey from Jasin (11.77 mg catechin/kg of honey) (Table 4). The variations in the flavonoid content could be due to the different honey types and their sources. Moniruzzaman et al. (2017) detected five flavonoids (catechin, myricetin, maringenin, hesperetin and kaempferol) in various Malaysian honeys. Polyphenols and flavonoids neutralize the reactive element by oxidising, resulting in a more stable, less-reactive radical. Therefore, honey containing higher polyphenols and flavonoid concentrations is preferred due to their antioxidant potential (Jaafar et al. 2017; Moniruzzaman et al. 2013b).

The antioxidant activity of unifloral honey samples was determined based on the scavenging activity against the free radical 2, 2-diphenyl-1-picryl-hydrazyl (DPPH). Among the several methods used to determine the antioxidant capacity of natural products, the nitrogen-based radical reagent DPPH has been extensively used to evaluate the radical-scavenging potential of honey (Mahnot et al. 2019; Di Marco et al. 2018; Alvarez-Suarez et al. 2010). In this study, the Jambul Merak (KPK) honey has the highest DPPH radical scavenging ability (67.66%), and its high radical scavenging activity might be due to its high phenolic and flavonoid contents (Table 4). Usually, a high DPPH scavenging activity confers high levels of antioxidant activity. Overall, the Jambul Merak honey has higher DPPH scavenging potential compared to previous reports such as Malaysian gelam, Borneo tropical honeys and Algerian honey (Moniruzzaman et al. 2013a; 2013b). Determination of specific antioxidant compounds that may be present in the stingless bees’ honey are recommended. Biluca et al. (2017) reported 26 phenolic compounds in honey from stingless bees. Similarly, Ranneh et al. (2018) found that Malaysian stingless bee honey had higher antioxidant activity compared to Tualang honey. This suggests strong association between the polyphenol content of stingless bee honey and antioxidant activities. Further work needs to be carried out to determine why stingless bees honey result in higher antioxidant activities.

## Conclusion

Analyses of the honey samples from 15 areas of West Coast of Malaysia revealed the occurrence of different types of pollens from 34 different plant families. Four honey samples were unifloral and the rest were multifloral. Predominant pollen of unifloral honey from Kedah (Kubang Pasu and Padang Terap districts) contained *Jacaranda obtusifolia* of *Bignoniaceae* family, from Jasin district of Melaka contained *Hevea brasiliensis* of *Euphorbiaceae* family and sample from Gertak Sanggul district of Penang contained *Cucumis melo* of *Cucurbitaceae* family. Malaysian honey can be recognized by the presence of coconut (*Cocos nucifera*) and palm oil (*Elaeis guineensis*) pollens, which are important food sources and are found to occur in almost every local honey and stingless bee honey samples. The antioxidant activity is high in Jambul Merak honey and it also contains higher color intensity, phenolic and flavonoid content indicating that there is a strong positive association between antioxidant capacity and phenolic and flavonoid content. The differences in physicochemical and antioxidant properties between the 3 unifloral honeys can be attributed to the origin of floral source. But the difference of properties between unifloral honey Jambul merak from different geographical locations show that the physicochemical and antioxidant properties are also dependent on geographical origin of the honeys.
